# Association between the Hypertriglyceridemic Waist Phenotype and Prediabetes in Chinese Adults Aged 40 Years and Older

**DOI:** 10.1155/2018/1031939

**Published:** 2018-06-25

**Authors:** Kun Zhao, Shan-Shan Yang, Hai-Bin Wang, Kang Chen, Zhao-Hui Lu, Yi-Ming Mu

**Affiliations:** ^1^Department of Endocrinology, Chinese PLA General Hospital, No. 28 Fuxing Road, Beijing 100853, China; ^2^Institute of Geriatrics, Beijing Key Laboratory of Ageing and Geriatrics, and State Key Laboratory of Kidney Disease, Chinese PLA General Hospital, No. 28 Fuxing Road, Beijing 100853, China

## Abstract

**Objective:**

To investigate the association between the hypertriglyceridemic waist (HTGW) phenotype and prediabetes in Chinese adults aged 40 years and older.

**Methods:**

12757 adults (4101 men and 8656 women) without diabetes or cardiovascular and cerebrovascular diseases, free of using lipid-modified agents, were enrolled in this cross-sectional study. The HTGW phenotype was defined as elevated serum triglyceride concentrations and enlarged waist circumference. A two-hour post 75 g oral glucose tolerance test was performed in all participants. Multiple logistic regression analysis was used to evaluate the relationship of the HTGW phenotype with prediabetes.

**Results:**

Individuals with the HTGW phenotype had a higher adjusted odds ratio (OR: 1.70; 95% CI: 1.48–1.95) of prediabetes than those without the phenotype. There existed a strong relationship of the HTGW phenotype with impaired glucose tolerance (IGT) (OR: 1.83; 95% CI: 1.57–2.13), but not with impaired fasting glucose (IFG) (OR: 0.87; 95% CI: 0.65–1.17). Only women with the HTGW phenotype are significantly associated with the combined IFG and IGT (OR: 1.83; 95% CI: 1.28–2.62).

**Conclusions:**

The HTGW phenotype was a useful risk indicator and a practical screening tool to benefit in the early diagnosis and intervention for prediabetes, particularly for IGT and the combined IFG and IGT.

## 1. Introduction

Diabetes mellitus, as one of the most common global public health problems, has raised serious concern due to its growing prevalence and incidence. According to the reference report of International Diabetes Federation (IDF), the number of people (aged 20–79 years) with diabetes worldwide was estimated to be 425 million in 2017 and may rise to 629 million in 2045. These numbers for Chinese diabetic adults were projected to be 114.4 million and 119.8 million, respectively [1]. However, it is notable that prediabetes, regarded as a disordered glucose metabolic status that remains below diabetes thresholds, has been much more prevalent than diabetes in China. The estimated prevalence of Chinese adults with prediabetes was 50.1%, and 493.4 million may have had prediabetes in 2010 [2]. Prediabetes is an important risk factor for diabetes [3] and many other diseases, such as cardiovascular diseases (CVD) [4], stroke [5], and nephropathy [6]. It was reported that the annual risk of progressing to diabetes in people with prediabetes was 5%–10% and up to 70% prediabetic patients would develop diabetes after years [7]. Nevertheless, most people with prediabetes are unaware of this metabolic abnormality and ignore its significance as they do not have overt symptoms. Hence, in order to promote early diagnosis and intervention of prediabetes, it is indispensable to find a simple, low-cost, and effective approach for screening individuals with prediabetes in an enormous population.

The hypertriglyceridemic-waist (HTGW) phenotype is characterized by the simultaneous presence of elevated fasting triglyceride (TG) concentrations and enlarged waist circumference (WC). Lemieux et al. [8] first verified that the HTGW phenotype could be used as an inexpensive screening tool to identify men characterized by the atherogenic metabolic triad (hyperinsulinemia, elevated apolipoprotein B, and small dense low-density lipoprotein-cholesterol particles) and at increased risk of coronary artery disease (CAD). So far, a few studies have demonstrated that the HTGW phenotype, in addition to its strong relationships with CVD [9], chronic kidney disease (CKD) [10], hypertension [11], and hyperuricemia [12], also played an important role in diabetes. Increasing evidence has shown a higher risk of diabetes in people with the HTGW phenotype and supported that the HTGW phenotype could be a robust predictor for diabetes [13–15]. However, only a few studies reported the relationship of the HTGW phenotype with prediabetes. Research conducted by Díaz-Santana et al. [16] indicated that the HTGW phenotype was strongly associated with prediabetes, but in the study of Chen et al. [17], there existed no association between the HGTW phenotype and prediabetes. Additionally, a cohort study in Chinese urban adults performed by Zhang et al. [18] revealed that the predictive effect of diabetes was only found in women with the HTGW phenotype, but not in men. Given the current controversy, in this cross-sectional study, we therefore aimed to investigate the association between the HTGW phenotype and prediabetes, including IFG, IGT, and combined IFG and IGT, among Chinese adults.

## 2. Design and Methods

### 2.1. Study Population

The present study, which was one part of the Risk Evaluation of cAncers in Chinese diabeTic Individuals: a lONgitudinal (REACTION) study (Clinical trial number NCT01506869), was conducted in three communities (Guchen, Jindingjie, and Laoshan) of Beijing from December 2011 to August 2012. A detailed description of the methods in the REACTION study has been published previously [19]. Individuals aged 40 years and older participate in the survey, with a required response rate of over 85%. A total number of 19443 people (6845 men and 12598 women) signed the informed consent form and were enrolled in this study. Of those, 12757 adults (4101 men and 8656 women) without diabetes or cardiovascular and cerebrovascular diseases, free of using lipid-modified agents, were included in the final analysis. The study protocol was approved by the Chinese People's Liberation Army General Hospital Ethics Committee.

### 2.2. Data Collection and Measurements

All the participants completed a detailed questionnaire and underwent the anthropometric and laboratory measurements. Data was collected by well-trained physicians with a standardized questionnaire, including demographic characteristics (i.e., age, gender, nation, and education), lifestyle (i.e., smoking, alcohol consumption, and physical activity), medical history (i.e., hypertension, coronary heart disease, myocardial infarction, and stroke), and family history of diabetes. Education was divided into four groups: (1) primary school, (2) middle school, (3) high school, and (4) college or above. Physical activity was estimated and classified as low, moderate and high based on the short version of the International Physical Activity Questionnaire (IPAQ).

The anthropometric information of all participants, including height, weight, waist circumference (WC), hip circumference (HC), systolic blood pressure (SBP), and diastolic blood pressures (DBP), were obtained by well-trained nurses. While participants were in light clothing and barefoot, weight was measured to the nearest 0.1 kg using a standard digital scale, and height, WC, and HC were measured to the nearest 0.1 cm using a nonelastic tape. WC was measured at the midpoint between the lowest rib and the top of the iliac crest. HC was measured around the widest part of the buttocks. Body mass index (BMI) was calculated as weight in kilograms divided by the square of height in meters (kg/m^2^). The waist-hip ratio (WHR) was calculated as WC divided by HC. Blood pressure was measured three times at two-minute intervals using an Omron HEM-9000AI device (OMRON, Omron Company, China) after participants rested for at least five minutes and seated in the position with the arm at the same level of the heart. SBP and DBP were calculated as the average of the three measurements.

All the blood samples were collected after an overnight fasting (at least ten hours), and then the participants underwent a two-hour post 75 g oral glucose tolerance test (OGTT). Fasting and two-hour postloading plasma glucose (FBG and 2 h-PG), glycated hemoglobin (HbA1c), serum triglyceride (TG), total cholesterol (TC), high-density lipoprotein cholesterol (HDL-C), and low-density lipoprotein cholesterol (LDL-C) were measured by an automatic analyzer (Cobas 8000 Modular Analyzer Series; Roche Diagnostics, Basel, Switzerland) in the laboratory of Chinese PLA General Hospital.

### 2.3. Definition of Variables

Participants were defined as never, former, or current smokers (who smoked at least one cigarette per day or seven cigarettes per week in the past 6 months) according to cigarette smoking habits. The type and frequency of alcohol consumptions were recorded, and never, former, or current alcohol drinking (consumed alcohol once per week regularly during the past 6 months) status was defined according to alcohol consumption habits [20, 21]. The family history of diabetes was defined as the diabetes history of parents, siblings, and children. According to the World Health Organization (WHO) criteria, overweight was defined as 25 ≤ BMI < 30 kg/m^2^ and obesity was defined as BMI ≥ 30 kg/m^2^. Hypertension was defined as a self-reported medical history of hypertension or SBP ≥ 140 mm Hg or DBP ≥ 90 mm Hg without any pharmacological intervention. Cardiovascular and cerebrovascular diseases were defined as a self-reported medical history of coronary heart disease, myocardial infarction, or stoke, which was excluded in this study.

Participants were categorized into four phenotype groups on the basis of the following cut-off points: (1) normal waist-normal triglycerides (NWNT): WC < 90 cm for men and < 80 cm for women and serum triglyceride concentration < 1.7 mmol/L; (2) normal waist-elevated triglycerides (HTG): WC < 90 cm for men and < 80 cm for women and serum triglyceride concentration ≥ 1.7 mmol/L; (3) enlarged waist-normal triglycerides (EW): WC ≥ 90 cm for men and ≥ 80 cm for women and serum triglyceride concentration < 1.7 mmol/L; and (4) hypertriglyceridemic waist (HTGW): WC ≥ 90 cm for men and ≥ 80 cm for women and serum triglyceride concentration ≥ 1.7 mmol/L. Based on the WHO criteria, prediabetes was divided into three subgroups as follows: (1) impaired fasting glucose (IFG): 6.1 ≤ FBG < 7.0 mmol/L and 2 h-PG < 7.8 mmol/L; (2) impaired glucose tolerance (IGT): FBG < 6.1 mmol/L and 7.8 ≤ 2 h-PG < 11.1 mmol/L; and (3) IFG + IGT: 6.1 ≤ FBG < 7.0 mmol/L and 7.8 ≤ 2 h-PG < 11.1 mmol/L. Diabetes was defined as self-reported diabetes and new-diagnosed diabetes (FBG ≥ 7.0 mmol/L or PBG ≥ 11.1 mmol/L), which was excluded in this study.

### 2.4. Statistical Analysis

All analyses were performed using SPSS for Windows (version 24.0; SPSS, Chicago, IL). Continuous and categorical variables were expressed as the mean ± standard deviation (SD) and *n* (%), respectively. An analysis of variance (ANOVA) test was used to compare continuous variables among different phenotype groups. The comparison of categorical data was performed by *χ*^2^-test analysis. Multiple logistic regression analysis was used to test the effects of four phenotype groups on prediabetes and its subgroups, and the results were presented as odds ratios (ORs) and 95% confidence intervals (CIs). Three multivariable models were built as follows: model 1—adjusted for age, gender, nation, and education; model 2—further adjusted for family history of diabetes, SBP, hypertension, TC, and HDL-c; and model 3—further adjusted for smoking, alcohol consumption, physical activity, and BMI. Two-tailed *P* values less than 0.05 (*P* < 0.05) were considered statistically significant.

## 3. Results

Table 1 presents the general characteristics of the 12757 participants in this cross-sectional study, including 4101 (32.1%) men and 8656 (67.9%) women, who were aged 40 years and older without diabetes or cardiovascular and cerebrovascular diseases, free of using lipid-modified agents. Among them, 3840 (30.1%) subjects were prediabetic, including 544 (4.3%) with IFG, 2608 (20.4%) with IGT, and 688 (5.4%) with both IFG and IGT. The numbers of all participants stratified by the 4 phenotype groups were 5089 (39.9%) in NWNT, 1277 (10.0%) in HTG, 4061 (31.8%) in EW, and 2330 (18.3%) in HTGW. Except race and family history of diabetes, other variables (age, gender, education, smoking, alcohol consumption, physical activity, BMI, WC, HC, WHR, SBP, DBP, hypertension, FPG, 2 h-PG, HbA1c, TC, TG, HDL-c, LDL-c, and prediabetes) exhibited a significant difference among the 4 phenotype groups (*P* < 0.01). Compared to those with the NWNT phenotype, participants with the HTGW phenotype were older and had a higher level of BMI, WC, HC, WHR, SBP, DBP, FPG, 2 h-PG, HbA1c, TC, TG, and LDL-c, but a lower level of HDL-c (*P* < 0.01).

According to the logistic regression analysis, Table 2 displays that in the total population, the HTGW phenotype was significantly associated with prediabetes in the unadjusted model (OR: 2.68; 95% CI: 2.41–2.98) and in multivariable models, including model 1 (OR: 2.63; 95% CI: 2.37–2.93), model 2 (OR: 2.04; 95% CI: 1.80–2.32), and model 3 (OR: 1.70; 95% CI: 1.48–1.95). In addition, the positive relationships between the HTGW phenotype and prediabetes were also shown in the men's group (OR: 1.57; 95% CI: 1.24–1.99) and women's group (OR: 1.75; 95% CI: 1.48–2.09).

Prediabetes was stratified by 3 subgroups, and the evaluations of the associations between the HTGW phenotype and IFG/IGT/IFG + IGT were displayed in Tables 3–5. After the adjustment for the multiple variables, the association between the HTGW phenotype and IFG reported no significant difference in the total population (OR: 0.87; 95% CI: 0.65–1.17), men's group (OR: 0.97; 95% CI: 0.63–1.50), or women's group (OR: 0.79; 95% CI: 0.53–1.19) (*P* > 0.05) (Table 3). However, in contrast to IFG, the results of IGT and IFG + IGT were similar to those of prediabetes.  Table 4 presents that participants with the HTGW phenotype suffered higher adjusted odds of IGT (OR: 1.83; 95% CI: 1.57–2.13) compared to those with the NWNT phenotype, and although the strength of the correlation between the HTGW phenotype and IGT was attenuated in multivariable models, it still remained significantly different (*P* < 0.01). The consistent results were also observed in the men's group (OR: 1.61; 95% CI: 1.23–2.12) and women's group (OR: 1.94; 95% CI: 1.61–2.34). Additionally, as shown in Table 5, after the adjustment for multiple variables, there still existed a strong relationship of the HTGW phenotype with the combined IFG and IGT in the total population (OR: 1.68; 95% CI: 1.28–2.21) and women's group (OR: 1.83; 95% CI: 1.28–2.62), but not in the men's group (OR: 1.52; 95% CI: 0.98–2.37).

**Figure 1:**
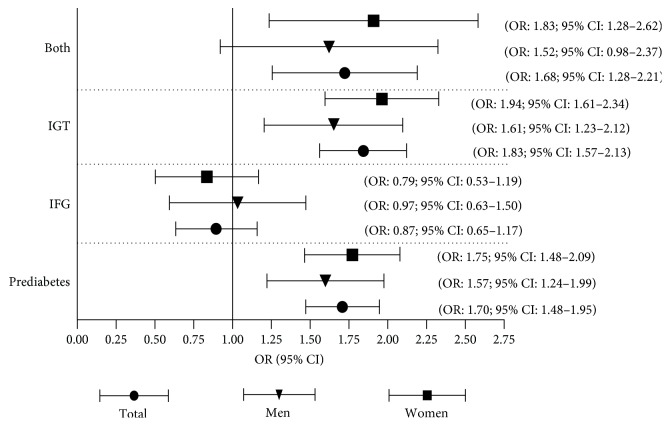
The associations between the HTGW phenotype and prediabetes/IFG/IGT/IFG+IGT in the total population, men's group, and women's group. Model 3: adjusted for age, gender, nation, education, family history of diabetes, SBP, hypertension, TC, HDL-c, smoking, alcohol consumption, physical activity, and BMI.

Based on the multiple logistic regression analysis mentioned above, Figure 1 is used to present the associations between the HTGW phenotype and prediabetes/IFG/IGT/IFG + IGT in the total population, men's group, and women's group, respectively. It showed the relationships of the HTGW phenotype with prediabetes/IGT/IFG + IGT were all stronger in the women's group than in the men's group, suggesting that women with the HTGW phenotype were more likely to develop prediabetes/IGT/IFG + IGT than men.

## 4. Discussion

It is well known that prediabetes is characterized by the hyperglycemic condition that includes IFG, IGT, or a combination of both [22]. The results of this cross-sectional study indicated that the HTGW phenotype was significantly associated with prediabetes in Chinese adults aged 40 years and older. Furthermore, it is noteworthy that the results varied in different subgroups of prediabetes.

Our present study showed a strong association between the HTGW phenotype and IGT, both in men and in women, which had not been reported before. In recent years, the HTGW phenotype has been considered a useful tool to distinguish visceral adiposity and subcutaneous adiposity, which was better than using an elevated WC alone [23, 24]. Meanwhile, researches showed that visceral adiposity was associated with IGT [25]. Accordingly, compared to the EW phenotype, the HTGW phenotype would be much more applicable to the identification of individuals with IGT. In the present study, however, no significant difference was observed in the relationship of the HTGW phenotype with IFG, which was consistent with the finding in Chen et al.'s study [17] but was opposed to other few researches. Yu et al. [26] and Díaz-Santana et al. [16] reported that individuals with the HTGW phenotype had higher adjusted odds of prediabetes/IFG than those without this phenotype in the Chinese population (men—OR: 2.1; 95% CI: 1.8–2.5; women—OR: 2.0; 95% CI: 1.6–2.3) and in the Hispanic population (OR: 5.55; 95% CI: 3.38–9.13), respectively. With regard to these discrepancies among studies, the reasons could be partially explained by the diverse definitions of the HTWG phenotype and the different criteria of IFG. Therefore, determining and using the most appropriate definitions and criteria based on ethnic- and gender-specific cutoff points would be crucial for more accurate analysis in further researches.

Moreover, our results demonstrated that there appeared a gender difference in the relationship between the HTGW phenotype and the combined IFG and IGT. After the adjustment for multiple variables, the strength of the association for both IFG and IGT remained statistically significant only in the women's group, but not in the men's group. The similar results were observed in the study of Zhang et al. [18], which indicated that the HTGW phenotype was strongly associated with the predicted development of prediabetes in women rather than in men. Additionally, in our previous research regarding the relationship of the HTGW phenotype with arterial stiffness, there also existed a sex disparity and the significant difference was only shown in the women's group [27]. The possible underlying mechanisms involved might result from the gender difference in the pattern of lipid metabolism, visceral fat accumulation, and body fat distribution [28, 29]. Hence, much more attention should be paid on women with the HTGW phenotype, since they would be more vulnerable to prediabetes than men.

There are several limitations in our study. First, the causal association of the HTGW phenotype and prediabetes was undetermined due to the nature of the cross-sectional study. Second, our sample may not be completely representative of all Chinese adults because the participants enrolled in this study were 40 years and older and lived in an urban district. Third, in spite of the adjustment for a wide range of potential confounders, there still existed possibilities that other unmeasured variables might be partially involved in the association between the HTGW phenotype and prediabetes. The strengths of this study include the large sample size and a 75 g OGTT that was performed in all subjects for the identification of IGT. To our best knowledge, this is the first article that provides novel evidence about the significant association between the HTGW phenotype and IGT, indicating that it is necessary to perform an OGTT rather than detecting FPG alone for the early recognition of prediabetes in individuals, particularly women, with the HTGW phenotype. Based on the merits and drawbacks mentioned above, further large-scaled prospective studies thus should be conducted to confirm the causal effect relationship of the HTGW phenotype with prediabetes and to give more insight into the predictive role of the HTGW phenotype in prediabetes.

## 5. Conclusion

In the present study, we drew the conclusions as follows: (1) the HTWG phenotype could be a useful risk indicator for the early diagnosis of individuals with IGT; (2) only women with the HTGW phenotype carried an increased odds ratio of the combined IFG and IGT; (3) there existed no significant difference between the HTGW phenotype and IFG. Considering the growing prevalence of prediabetes, it is essential to raise public awareness about the early recognition and intervention of prediabetes, which at the same time contribute to preventing the progression of diabetes and further conduce to the reduction of other relevant diseases, particularly CVD. Thus, the HTGW phenotype, an early warning sign, should be recommended to be widely used as an inexpensive and efficient screening tool for the identification of individuals at a high risk of prediabetes in future clinical practice and epidemiological studies.

## Figures and Tables

**Table 1:** Characteristics of the participants in four phenotype groups (*n* = 12757).

Variables	NWNT	HTG	EW	HTGW	Total	*P* value
	(*N* = 5089)	(*N* = 1277)	(*N* = 4061)	(*N* = 2330)	(*N* = 12757)	
Age (year)	55.3 ± 7.9	55.2 ± 7.1	56.2 ± 7.8^∗^	56.4 ± 7.2^∗^	55.8 ± 7.7	<0.001
Gender, *n* (%)						<0.001
Men	1909 (37.5)	672 (52.6)	864 (21.3)	656 (28.2)	4101 (32.1)	
Women	3180 (62.5)	605 (47.4)	3197 (78.7)	1674 (71.8)	8656 (67.9)	
Race, *n* (%)						0.644
Han	4858 (95.5)	1218 (95.4)	3852 (94.9)	2206 (94.7)	12134 (95.1)	
Others	231 (4.5)	59 (4.6)	209 (5.1)	124 (5.3)	623 (4.9)	
Educational status, *n* (%)						<0.001
Primary school or below	209 (4.1)	42 (3.3)	366 (9.0)	142 (6.1)	759 (5.9)	
Middle school	1463 (28.7)	378 (29.6)	1381 (34.0)	829 (35.6)	4051 (31.8)	
High school	2436 (47.9)	599 (46.9)	1751 (43.1)	1012 (43.5)	5798 (45.5)	
College or above	981 (19.3)	258 (20.2)	563 (13.9)	347 (14.9)	2149 (16.8)	
Current smokers, *n* (%)	933 (18.4)	384 (30.1)	443 (10.9)	413 (17.8)	2173 (17.0)	<0.001
Current drinker, *n* (%)	572 (11.3)	223 (17.5)	315 (7.8)	210 (9.0)	1320 (10.3)	<0.001
Physical activity, *n* (%)						0.002
Low	942 (18.5)	248 (19.5)	666 (16.4)	454 (19.5)	2310 (18.1)	
Moderate	3538 (69.5)	888 (69.5)	2930 (72.1)	1647 (70.7)	9003 (70.6)	
High	609 (12.0)	141 (11.0)	465 (11.5)	229 (9.8)	1444 (11.3)	
Family history of diabetes, *n* (%)	935 (18.4)	243 (19.0)	738 (18.2)	400 (17.2)	2316 (18.2)	0.301
BMI (kg/m^2^)	23.2 ± 2.5	24.3 ± 2.3^∗^	27.3 ± 3.1^∗^	27.8 ± 2.9^∗^	25.4 ± 3.4	<0.001
Overweight, *n* (%)	1797 (35.3)	637 (49.9)	2093 (51.5)	1146 (49.2)	5673 (44.5)	<0.001
Obesity, *n* (%)	103 (2.0)	65 (5.1)	1477 (36.4)	1008 (43.3)	2653 (20.8)	<0.001
WC (cm)	76.3 ± 6.4	79.5 ± 5.9^∗^	88.2 ± 6.8^∗^	89.8 ± 6.8^∗^	82.9 ± 8.9	<0.001
HC (cm)	90.2 ± 5.1	91.1 ± 4.9^∗^	97.9 ± 6.3^∗^	98.4 ± 6.2^∗^	94.3 ± 6.9	<0.001
WHR	0.8 ± 0.1	0.9 ± 0.1^∗^	0.9 ± 0.1^∗^	0.9 ± 0.1^∗^	0.9 ± 0.1	<0.001
SBP (mmHg)	126.8 ± 16.1	131.5 ± 15.7^∗^	131.5 ± 16.5^∗^	134.1 ± 16.2^∗^	130.0 ± 16.4	<0.001
DBP (mmHg)	73.6 ± 9.4	77.1 ± 9.7^∗^	76.1 ± 9.8^∗^	78.4 ± 9.3^∗^	75.6 ± 9.7	<0.001
Hypertension, *n* (%)	851 (16.7)	258 (20.2)	1172 (28.9)	762 (32.7)	3043 (23.9)	<0.001
FPG (mmol/L)	5.3 ± 0.5	5.4 ± 0.5^∗^	5.4 ± 0.5^∗^	5.5 ± 0.5^∗^	5.4 ± 0.5	<0.001
2 h-PG (mmol/L)	6.4 ± 1.5	6.9 ± 1.7^∗^	7.0 ± 1.6^∗^	7.3 ± 1.6^∗^	6.8 ± 1.7	<0.001
HbA1c (%)	5.7 ± 0.5	5.8 ± 0.5^∗^	5.8 ± 0.4^∗^	5.8 ± 0.5^∗^	5.8 ± 0.5	<0.001
Prediabetes, *n* (%)	1089 (21.4)	435 (34.1)	1334 (32.8)	982 (42.1)	3840 (30.1)	<0.001
IFG, *n* (%)	203 (3.9)	61 (4.8)	162 (4.0)	118 (5.1)	544 (4.3)	
IGT, *n* (%)	740 (14.5)	278 (21.8)	929 (22.9)	661 (28.4)	2608 (20.4)	
IFG + IGT, *n* (%)	146 (2.9)	96 (7.5)	243 (5.9)	203 (8.7)	688 (5.4)	
TC (mmol/L)	5.1 ± 1.5	5.7 ± 1.8^∗^	5.2 ± 0.9	5.6 ± 1.0^∗^	5.3 ± 1.3	<0.001
TG (mmol/L)	1.0 ± 0.3	2.8 ± 1.7^∗^	1.1 ± 0.3^∗^	2.7 ± 1.3^∗^	1.5 ± 1.1	<0.001
HDL-c (mmol/L)	1.6 ± 0.5	1.2 ± 0.3^∗^	1.5 ± 0.4^∗^	1.2 ± 0.3^∗^	1.5 ± 0.4	<0.001
LDL-c (mmol/L)	3.1 ± 0.8	3.4 ± 0.9^∗^	3.2 ± 0.7^∗^	3.5 ± 0.8^∗^	3.2 ± 0.8	<0.001

Values are expressed as the mean ± SD or *n* (%). NWNT: normal waist-normal triglycerides; HTG: normal waist-elevated triglycerides; EW: enlarged waist-normal triglycerides; HTGW: hypertriglyceridemic waist; FHD: family history of diabetes; BMI: body mass index; WC: waist circumference; HC: hip circumference; WHR: waist-hip ratio; SBP: systolic blood pressure; DBP: diastolic blood pressure; FBG: fasting blood glucose; 2 h-PG: two-hour postloading plasma glucose blood glucose; HbA1c: hemoglobin A1c; TC: total cholesterol; TG: triglyceride; HDL-C: high-density lipoprotein cholesterol; LDL-C: low-density lipoprotein cholesterol. ^∗^*P* < 0.01 compared with NWNT.

**Table 2:** Logistic regression analysis assessing the associations between the HTGW phenotype and prediabetes for men and women.

Phenotype groups	Unadjusted			Model 1			Model 2			Model 3		
	OR	95% CI	*P*	OR	95% CI	*P*	OR	95% CI	*P*	OR	95% CI	*P*
*Total*												
NWNT	1.00			1.00			1.00			1.00		
HTG	1.88	1.65–2.15	<0.001^∗^	1.92	1.67–2.19	<0.001^∗^	1.59	1.37–1.85	<0.001^∗^	1.59	1.37–1.84	<0.001^∗^
EW	1.80	1.64–1.97	<0.001^∗^	1.76	1.60–1.94	<0.001^∗^	1.55	1.41–1.72	<0.001^∗^	1.27	1.13–1.42	<0.001^∗^
HTGW	2.68	2.41–2.98	<0.001^∗^	2.63	2.37–2.93	<0.001^∗^	2.04	1.80–2.32	<0.001^∗^	1.70	1.48–1.95	<0.001^∗^
*Men*												
NWNT	1.00			1.00			1.00			1.00		
HTG	1.45	1.20–1.76	<0.001^∗^	1.67	1.37–2.03	<0.001^∗^	1.51	1.22–1.86	<0.001^∗^	1.49	1.20–1.84	<0.001^∗^
EW	1.56	1.31–1.86	<0.001^∗^	1.60	1.34–1.90	<0.001^∗^	1.43	1.20–1.72	<0.001^∗^	1.19	0.96–1.47	0.111
HTGW	1.86	1.54–2.24	<0.001^∗^	2.13	1.76–2.58	<0.001^∗^	1.80	1.46–2.22	<0.001^∗^	1.57	1.24–1.99	<0.001^∗^
*Women*												
NWNT	1.00			1.00			1.00			1.00		
HTG	2.26	1.87–2.74	<0.001^∗^	2.18	1.80–2.64	<0.001^∗^	1.69	1.37–2.08	<0.001^∗^	1.69	1.37–2.08	<0.001^∗^
EW	2.06	1.83–2.31	<0.001^∗^	1.90	1.69–2.14	<0.001^∗^	1.65	1.46–1.86	<0.001^∗^	1.33	1.15–1.53	<0.001^∗^
HTGW	3.30	2.89–3.76	<0.001^∗^	2.98	2.60–3.40	<0.001^∗^	2.16	1.85–2.54	<0.001^∗^	1.75	1.48–2.09	<0.001^∗^

NWNT: normal waist-normal triglycerides; HTG: normal waist-elevated triglycerides; EW: enlarged waist-normal triglycerides; HTGW: hypertriglyceridemic waist. Model 1: adjusted for age, gender, nation, and education. Model 2: adjusted for Model 1, family history of diabetes, SBP, hypertension, TC, and HDL-c. Model 3: adjusted for Model 2, smoking, alcohol consumption, physical activity, and BMI. ^∗^*P* < 0.01 compared with NWNT.

**Table 3:** Logistic regression analysis assessing the associations between the HTGW phenotype and IFG for men and women.

Phenotype groups	Unadjusted			Model 1			Model 2			Model 3		
	OR	95% CI	*P*	OR	95% CI	*P*	OR	95% CI	*P*	OR	95% CI	*P*
*Total*												
NWNT	1.00			1.00			1.00			1.00		
HTG	1.21	0.90–1.62	0.207	1.12	0.83–1.50	0.464	0.99	0.72–1.35	0.943	0.95	0.69–1.30	0.823
EW	1.00	0.81–1.23	0.999	1.08	0.87–1.34	0.480	0.99	0.79–1.23	0.918	0.77	0.60–1.00	0.052
HTGW	1.28	1.02–1.62	0.035#	1.34	1.06–1.69	0.015#	1.12	0.86–1.45	0.408	0.87	0.65–1.17	0.353
*Men*												
NWNT	1.00			1.00			1.00			1.00		
HTG	0.93	0.63–1.38	0.733	0.93	0.63–1.37	0.696	0.84	0.55–1.26	0.392	0.82	0.54–1.24	0.339
EW	0.86	0.60–1.24	0.433	0.85	0.59–1.23	0.396	0.78	0.54–1.13	0.190	0.64	0.41–0.98	0.041#
HTGW	1.36	0.96–1.93	0.081	1.33	0.93–1.88	0.115	1.12	0.77–1.54	0.551	0.97	0.63–1.50	0.899
*Women*												
NWNT	1.00			1.00			1.00			1.00		
HTG	1.42	0.90–2.22	0.130	1.39	0.89–2.18	0.152	1.18	0.73–1.89	0.503	1.12	0.70–1.82	0.632
EW	1.27	0.96–1.67	0.089	1.22	0.92–1.61	0.169	1.10	0.83–1.47	0.499	0.85	0.60–1.18	0.329
HTGW	1.39	1.01–1.91	0.042#	1.33	0.96–1.84	0.082	1.07	0.74–1.54	0.720	0.79	0.53–1.19	0.259

NWNT: normal waist-normal triglycerides; HTG: normal waist-elevated triglycerides; EW: enlarged waist-normal triglycerides; HTGW: hypertriglyceridemic waist. Model 1: adjusted for age, gender, nation, and education. Model 2: adjusted for model 1, family history of diabetes, SBP, hypertension, TC, and HDL-c. Model 3: adjusted for model 2, smoking, alcohol consumption, physical activity, and BMI. ^#^*P* < 0.05 compared with NWNT.

**Table 4:** Logistic regression analysis assessing the associations between the HTGW phenotype and IGT for men and women.

Phenotype groups	Unadjusted			Model 1			Model 2			Model 3		
	OR	95% CI	*P*	OR	95% CI	*P*	OR	95% CI	*P*	OR	95% CI	*P*
*Total*												
NWNT	1.00			1.00			1.00			1.00		
HTG	1.64	1.40–1.91	<0.001^∗^	1.69	1.45–1.98	<0.001^∗^	1.53	1.30–1.81	<0.001^∗^	1.55	1.31–1.83	<0.001^∗^
EW	1.74	1.57–1.94	<0.001^∗^	1.66	1.49–1.86	<0.001^∗^	1.53	1.36–1.71	<0.001^∗^	1.42	1.25–1.62	<0.001^∗^
HTGW	2.33	2.07–2.62	<0.001^∗^	2.24	1.98–2.53	<0.001^∗^	1.93	1.68–2.21	<0.001^∗^	1.83	1.57–2.13	<0.001^∗^
*Men*												
NWNT	1.00			1			1.00			1.00		
HTG	1.39	1.11–1.73	0.003^∗^	1.61	1.29–2.03	<0.001^∗^	1.58	1.25–2.01	<0.001^∗^	1.61	1.26–2.05	<0.001^∗^
EW	1.55	1.27–1.89	<0.001^∗^	1.59	1.30–1.95	<0.001^∗^	1.50	1.21–1.84	<0.001^∗^	1.45	1.13–1.85	<0.001^∗^
HTGW	1.47	1.18–1.83	<0.001^∗^	1.71	1.36–2.15	<0.001^∗^	1.62	1.27–2.06	<0.001^∗^	1.61	1.23–2.12	<0.001^∗^
*Women*												
NWNT	1.00			1			1.00			1.00		
HTG	1.85	1.49–2.30	<0.001^∗^	1.78	1.43–2.21	<0.001^∗^	1.52	1.21–1.91	<0.001^∗^	1.53	1.21–1.93	<0.001^∗^
EW	1.89	1.66–2.15	<0.001^∗^	1.76	1.54–2.01	<0.001^∗^	1.59	1.39–1.82	<0.001^∗^	1.46	1.25–1.71	<0.001^∗^
HTGW	2.84	2.46–3.29	<0.001^∗^	2.58	2.22–2.98	<0.001^∗^	2.08	1.76–2.47	<0.001^∗^	1.94	1.61–2.34	<0.001^∗^

NWNT: normal waist-normal triglycerides; HTG: normal waist-elevated triglycerides; EW: enlarged waist-normal triglycerides; HTGW: hypertriglyceridemic waist. Model 1: adjusted for age, gender, nation, and education. Model 2: adjusted for model 1, family history of diabetes, SBP, hypertension, TC, and HDL-c. Model 3: adjusted for model 2, smoking, alcohol consumption, physical activity, and BMI. ^∗^*P* < 0.01 compared with NWNT.

**Table 5:** Logistic regression analysis assessing the associations between the HTGW phenotype and IFG + IGT for men and women.

Phenotype groups	Unadjusted			Model 1			Model 2			Model 3		
	OR	95% CI	*P*	OR	95% CI	*P*	OR	95% CI	*P*	OR	95% CI	*P*
*Total*												
NWNT	1.00			1.00			1.00			1.00		
HTG	2.69	2.06–3.51	<0.001^∗^	2.69	2.06–3.52	<0.001^∗^	2.16	1.63–2.88	<0.001^∗^	2.07	1.55–2.76	<0.001^∗^
EW	2.15	1.75–2.66	<0.001^∗^	2.15	1.74–2.67	<0.001^∗^	1.83	1.47–2.29	<0.001^∗^	1.27	0.99–1.63	0.059
HTGW	3.23	2.60–4.02	<0.001^∗^	3.24	2.59–4.04	<0.001^∗^	2.38	1.86–3.06	<0.001^∗^	1.68	1.28–2.21	<0.001^∗^
*Men*												
NWNT	1.00			1.00			1.00			1.00		
HTG	1.89	1.26–2.67	<0.001^∗^	2.10	1.43–3.08	<0.001^∗^	1.68	1.11–2.55	0.015#	1.59	1.05–2.42	0.030#
EW	1.91	1.31–2.62	<0.001^∗^	1.96	1.38–2.79	<0.001^∗^	1.65	1.15–2.38	0.001^∗^	1.12	0.74–1.71	0.586
HTGW	2.63	1.75–3.54	<0.001^∗^	2.89	2.03-4.13	<0.001^∗^	2.11	1.41–3.16	<0.001^∗^	1.52	0.98–2.37	0.061
*Women*												
NWNT	1.00			1.00			1.00			1.00		
HTG	3.58	2.46–5.23	<0.001^∗^	3.47	2.38–5.06	<0.001^∗^	2.80	1.87–4.17	<0.001^∗^	2.75	1.84–4.10	<0.001^∗^
EW	2.57	1.95–3.39	<0.001^∗^	2.37	1.79–3.14	<0.001^∗^	2.04	1.53–2.72	<0.001^∗^	1.40	1.02–1.93	0.040#
HTGW	3.91	2.93–5.23	<0.001^∗^	3.57	2.66–4.78	<0.001^∗^	2.65	1.91–3.69	<0.001^∗^	1.83	1.28–2.62	0.001^∗^

NWNT: normal waist-normal triglycerides; HTG: normal waist-elevated triglycerides; EW: enlarged waist-normal triglycerides; HTGW: hypertriglyceridemic waist. Model 1: adjusted for age, gender, nation, and education. Model 2: adjusted for model 1, family history of diabetes, SBP, hypertension, TC, and HDL-c. Model 3: adjusted for model 2, smoking, alcohol consumption, physical activity, and BMI. ^#^*P* < 0.05 compared with NWNT. ^∗^*P* < 0.01 compared with NWNT.

## Data Availability

The datasets used to support this study are not freely available in view of participants' privacy protection.
